# Real-Time Impact Visualization Inspection of Aerospace Composite Structures with Distributed Sensors

**DOI:** 10.3390/s150716536

**Published:** 2015-07-08

**Authors:** Liang Si, Horst Baier

**Affiliations:** Institute of Lightweight Structures, Faculty of Mechanical Engineering, Technische Universität München, Boltzmannstr. 15, Garching 85748, Germany; E-Mail: baier@tum.de

**Keywords:** real time, impact monitoring, hybrid thresholding filter, fast genetic algorithm, parameter estimation, composite structures, random vibration noises, structural state awareness

## Abstract

For the future design of smart aerospace structures, the development and application of a reliable, real-time and automatic monitoring and diagnostic technique is essential. Thus, with distributed sensor networks, a real-time automatic structural health monitoring (SHM) technique is designed and investigated to monitor and predict the locations and force magnitudes of unforeseen foreign impacts on composite structures and to estimate in real time mode the structural state when impacts occur. The proposed smart impact visualization inspection (IVI) technique mainly consists of five functional modules, which are the signal data preprocessing (SDP), the forward model generator (FMG), the impact positioning calculator (IPC), the inverse model operator (IMO) and structural state estimator (SSE). With regard to the verification of the practicality of the proposed IVI technique, various structure configurations are considered, which are a normal CFRP panel and another CFRP panel with “orange peel” surfaces and a cutout hole. Additionally, since robustness against several background disturbances is also an essential criterion for practical engineering demands, investigations and experimental tests are carried out under random vibration interfering noise (RVIN) conditions. The accuracy of the predictions for unknown impact events on composite structures using the IVI technique is validated under various structure configurations and under changing environmental conditions. The evaluated errors all fall well within a satisfactory limit range. Furthermore, it is concluded that the IVI technique is applicable for impact monitoring, diagnosis and assessment of aerospace composite structures in complex practical engineering environments.

## 1. Introduction

The use of composite materials in aerospace and automotive applications is increasing consistently due to their high specific strength, light weight, resistance to fatigue/corrosion and design flexibility. Typical application examples include the latest Airbus A380 aircraft that is 25% composite materials, and Airbus will be the first manufacturer to build an aircraft with an all-composite wing in their latest A350-XWB model and its entire structure will be composed of 45% composite materials. However, the mechanical properties of composite materials may degrade severely if damaged. This damage may be caused by imperfections introduced during the manufacturing process or by external loads during the operational life, such as impacts by foreign objects. Damages due to impact such as matrix cracking, delamination and fiber breakage, significantly reduces the structural integrity of the composite structure. Some conventional Non-Destructive Testing (NDT) detection solutions are very difficult to implement for aerospace transportation vehicles to monitor and diagnose damage online and on board. There exist some obvious shortcomings for the NDT technique, for instance, (1) the NDT instruments can’t be applied or attached permanently to the aerospace structure (or parts of the structure); (2) manual operation is necessary for the NDT technique, making it is costly, labor-intensive and time-consuming; (3) the NDT technique requires the removal of installed parts, which precludes quick inspection in hazardous areas (e.g., the fuel tank area) and congested areas. Therefore, an efficient technique that can automatically monitor and report events’ occurrences, their locations and force magnitudes and any structural state changes as a result of impacts would be very helpful at reducing the maintenance cost of large-scale aerospace structures.

In recent years, there has been an extensive amount of research associated with the development of multi-fusion Non-Destructive Testing management [1] and health monitoring methods for aerospace structure systems, but most investigations [2,3,4,5,6,7] have only taken into account their tested structure systems under “Perfectly Impracticable Environment Conditions” without any interfering factor, for instance, the existing background (interfering) noises from the mutative vibration environments of practical aerospace engineering. However, in case the impact visualization inspection technique developed could be applied successfully to implement efficiently impact positioning, identification and structural state assessment due to unforeseen impact events under various structure configurations and the disturbance factor that is random interfering noises from structural vibration, it would be a very significant step toward solving practical aerospace engineering problems.

Qiu and Yuan [8,9] used a time reversal-based impact positioning method to monitor impact events on complex aircraft wing structures. Nevertheless, a new environmental disturbance factor and mutative structure configurations are proposed in this study, and the novel impact visualization inspection technique is developed to establish more accurate forward and inverse models for monitoring any impact event on structures. The forward model is constructed in terms of impulse response functions (IRFs) on the basis of the relation between input and output, which can be applicable to various structure configurations and can also handle various types of impact objects. Meanwhile, because it provides simple inverse model formulations to identify input forces, thus the entire impact identification procedure become much simpler and faster than the model-based identification technique [10,11,12,13,14,15,16,17]. To interpret the whole impact visualization inspection procedures, they are presented from two major aspects in this paper, which are theoretical development and experimental verification. In the impact visualization inspection technique, a theoretical basis is found using the simulated output data to determine unknown impacts, which are based on the sensor measurements of structural responses. In the subsequent sections describing experimental tests and results and discussion, the developed impact visualization inspection technique is verified and assessed by a series of relevant impact experiments.

## 2. Method of Approach

**Figure 1:**
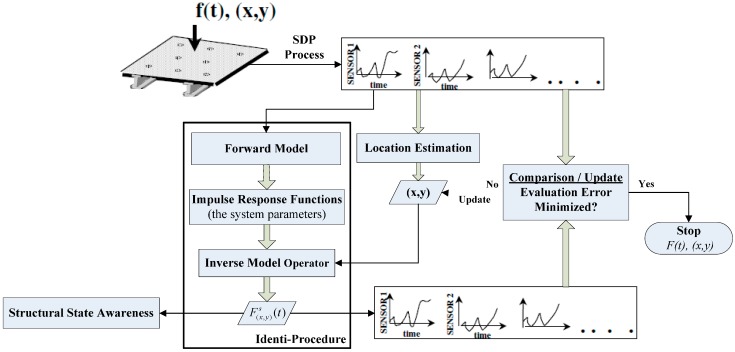
Overview of the automatic implementation procedure of the IVI technique.

This developed IVI approach is an automatic inspection technique based on global sensor measurements as illustrated in Figure 1, which is adaptable to various structure configurations and various types of impact objects. However, whatever the traditional mechanical modeling approach used—model-based identification techniques or neural networks-based identification technique, they implement location and reconstruction of impact force based on local sensor measurements, and they have their own irreparable flaws. In view of the above background, the methodology and advantages of the IVI technique will be described in the following sections.

### 2.1. Signal Data Preprocessing

In order to de-noise the original sensor signals, a mode decomposition-based filtering method—the real-time empirical mode decomposition (EMD, [18])-based hybrid thresholding filter [19,20] is adopted to eliminate the interferences (e.g., random interfering noises) and transfer smoothly the nonlinear effects from non-stationary output signals due to the weak vibration to the linear dependence; that is, discovering and extracting the effective linear relation between the input and the output from the structural response subject to sudden impact, which is hidden in the nonlinear condition.

**Figure 2:**
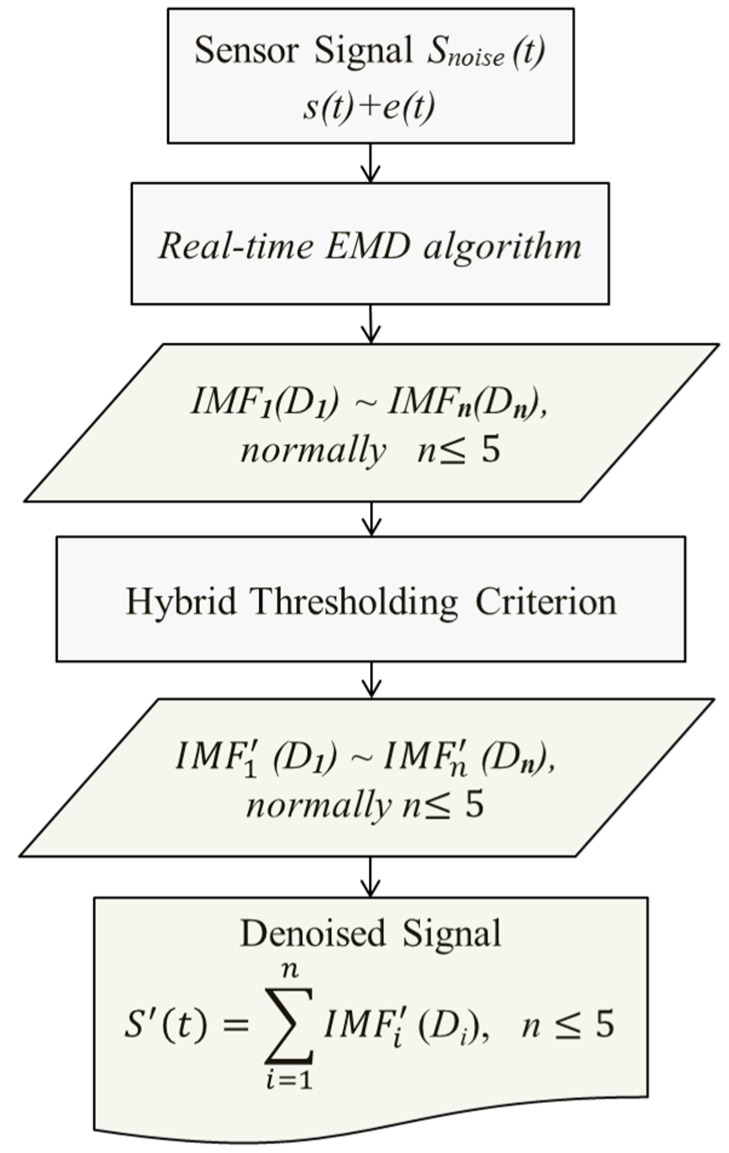
Flowchart of the real-time EMD based hybrid thresholding filtering process.

The SDP procedure based on real-time EMD-HTF (as shown in Figure 2) is as follows:

(1) Temporal sensor signals S(t) with random noises are decomposed by real-time EMD;
(2) From the scales with the valuable information, the first five useful scales (such as 1st, 2nd, 3rd, 4th, 5th scales) are extracted normally, and then choose an appropriate threshold at every scale and remove the interfering noises using Equation (1), defined as: 
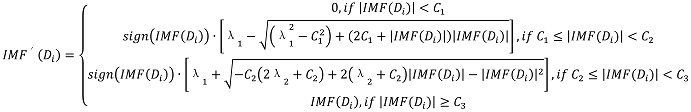
(1) where $C_{2}$ is the threshold which will be determined by a kurtosis-based approach individually for every scale and is given by $C_{2}=\frac{\text{max}\left(\left|Z\right|\right)}{Kurt\left(Z\right)/3}$, Z is the IMF coefficient of a scale, Kurt(Z) is the kurtosis of the decomposed components, $C_{1}$, $C_{3}$, ${\text{λ}}_{1}\;$ and ${\text{λ}}_{2}\;$ will be specified according to the $C_{2}\;$ value in each scale.

**Figure 3:**
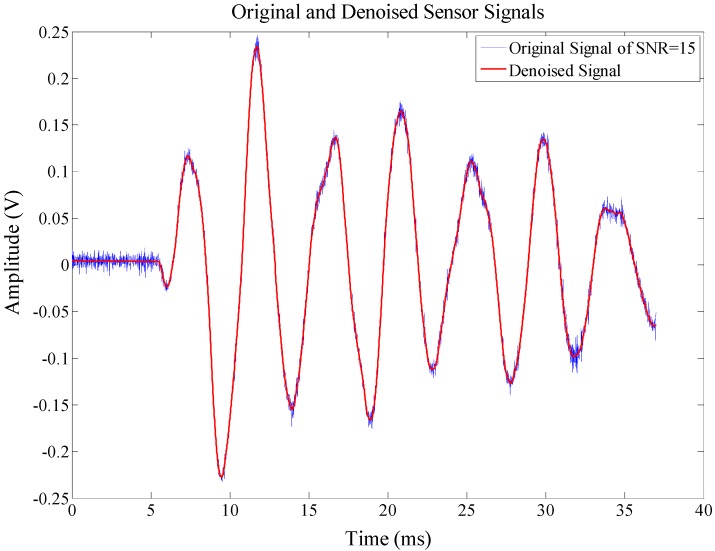
A sensor output signal from the structural response within noise.

The resulting intrinsic mode function (IMF) coefficients, mainly from the first five scales, are combined and reconstructed. Finally, the filtered signal can be obtained from the reconstruction process, which is illustrated in Figure 3.

### 2.2. Impact Identification Procedure

#### 2.2.1. Forward Model

In the procedure of establishing an accurate forward model, three main functional modules are executed, as follows:

(1) Determination of the forward system model structure;
(2) The optimal model order selection based on prediction error minimization (PEM);
(3) Generation of the optimal parameters ($a_{i},b_{j}$) of the model structure and formation of impulse response function matrices.

In the first functional module of system model structure determination, the goal is to determine a suitable forward system model. Then the built forward system model provides a basis to perform the next procedure of the optimal model order selection.

##### Fast Genetic Algorithm Parameter Estimation Assisted Impulse Response Function Matrices Generator

To minimize the prediction errors between the real outputs and the modeled outputs and obtain the simulated optimized forward outputs, and further obtain the impulse response function matrix from the calculated system parameters ($a_{i},\;b_{j}$), a fast Genetic Algorithm [21] parameter estimation (FGAPE) method [22] is defined and applied to the calculation procedure of solving the values of ($a_{i},\;b_{j}$) from the finalized forward system model, by rapidly training the needed data.

For fast GA-based error estimation, there are normally five steps that need to be executed, which are: (1) Reproduction; (2) Crossover; (3) Mutation; (4) New blood; (5) Elite. However, a multi-entropy $H\left(a_{n},\;b_{m}\right)$ shown in Equation (2) has to be defined to minimize the prediction error through the proposed fast GAPE training method:

(2) $$\tilde{\theta }{\left[a_{n},\;b_{m}\right]}_{opt}=H\left(a_{n},\;b_{m}\right)\;$$

where the entropy $H\left(a_{n},\;b_{m}\right)$ is decomposed into two factors $H\left(a_{n}\right)$ and $H\left(b_{m}\right)$ shown in Equations (3) and (4):

(3) $$H\left(a_{n}\right)=-\overset{n}{\underset{i=1}{\sum }}P(a_{n})\log P\left(a_{n}\right)$$

(4) $$H\left(b_{m}\right)=-\overset{m}{\underset{i=1}{\sum }}P(b_{m})\log P\left(b_{m}\right)$$

Finally, the IRF matrix $G_{f}^{s}$ (shown as Equation (5)) demanded can be obtained:

(5) $$G_{f}^{s}=\left[\begin{array}{ccccc} g\left(0\right) & 0 & 0 & \Lambda & 0\\ g\left(1\right) & g\left(0\right) & 0 & \Lambda & 0\\ M & M & O & 0 & M\\ g\left(n-2\right) & g\left(n-3\right) & O & O & 0\\ g\left(n-1\right) & g\left(n-2\right) & \Lambda & g\left(1\right) & g\left(0\right) \end{array}\right]\;$$

However, the impulse response s(k) of a structure system can be represented in a matrix convolution formulation given by Equation (6):

(6) $$S_{\left(x^{\prime },y^{\prime }\right)}=G_{f}^{s}U_{\left(x,y\right)}\;$$

**Figure 4:**
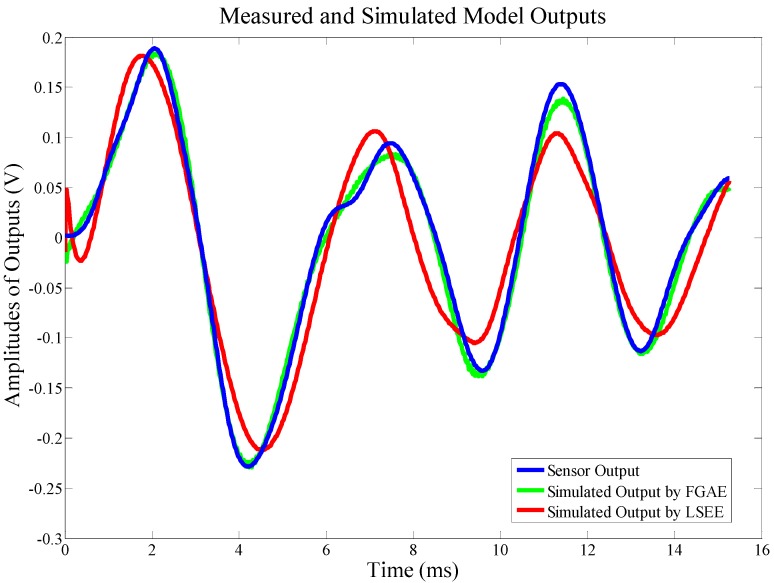
An example of forward model verification.

In that way, in order to indicate the accuracy of the forward system model simulated through fast GA parameter estimation, the simulation results are compared with recorded sensor signals and the modeled output using least squares error (LSE) estimation, shown in Figure 4. This figure reveals intuitively that the accuracy of the simulating output from FGAPE is much better than that of LSEE.

#### 2.2.2. Inverse Model Operator

In order to reconstruct impact forces, the force signals from impacts can be predicted based on the inverse model operator using the output data of the structure responses. Then, by applying the structure system model found and the impulse response expression (Equation (6)) described in Section 2.2.1, the inverse IR model for force reconstruction can be found and the corresponding force signals due to impacts can be also calculated by this inverse procedure.

Based on the assumption of zero initial conditions, the inverse IR model can be established as follows:

(7) $$\text{u}\left(\text{k}\right)=\overset{k}{\underset{i=0}{\sum }}\hat{g}\left(i\right)s\left(k+r-i\right)\;k=1,\;2,\;\ldots \;$$

where the inverse impulse response function matrices are defined by:

(8) $$\hat{g}\left(0\right)={\left(CA^{r-1}B\right)}^{T}\;\hat{g}\left(i\right)=\hat{C}{\hat{A}}^{i-1}\hat{B}\;i=1,\;2,\;\ldots$$

Finally, an impact force which is applied at the location (x, y) on a structure can be reconstructed through Equation (9) that is the matrix convolution expression of Equation (7): 
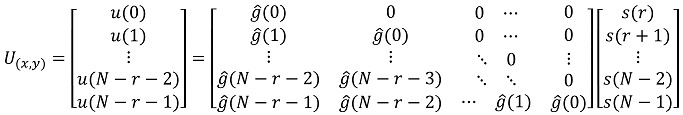
(9)

**Figure 5:**
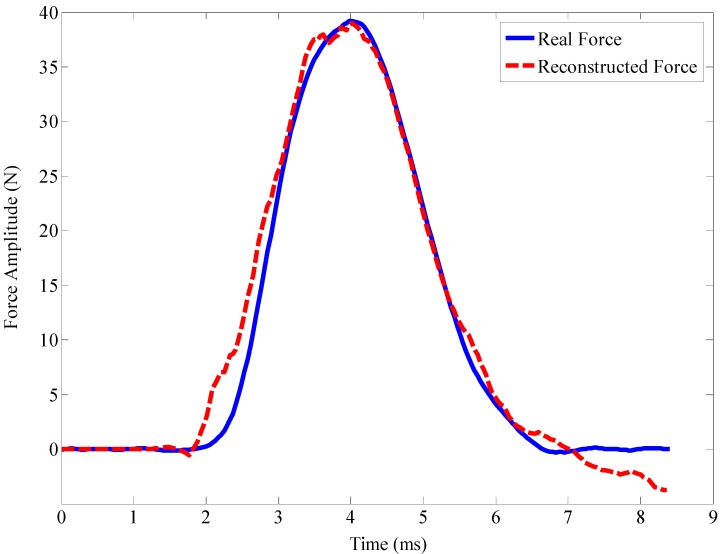
Demonstration of an impact force reconstruction.

An example of force reconstruction is illustrated in Figure 5.

### 2.3. Impact Positioning Calculation

To determine multi-impact locations and decrease the estimation time for impact location and reconstruction, an initial estimation method needs to be adopted. Therefore, a smooth power distribution method is proposed to search and identify the sensors closest to the impact points, which is a rapid and accurate way to locate the impacted regions without the susceptibility resulting from any measurement noise. This method allows one or multiple sensor array regions to be formed and isolated, that afterwards are distributed to update the accurate locations of impact forces.

#### 2.3.1. Extraction of Impact Region

By comparing the signal powers in the chosen sensor data time windows, the closest sensor arrays are determined and the corresponding powers from the sensor signals can be calculated as:

(10) $$P=\frac{1}{t_{1}-t_{0}}\overset{t_{1}}{\underset{i=t_{0}}{\sum }}{\left|s_{exp}\left(i\right)\right|}^{2}$$

where *P* is the signal power of the each sensor interested; $t_{0}$ is the initial time point, and $t_{1}$ is the final time point chosen. The time window of each sensor signal should be selected so that the condition that a sensor array close to each impact has a large power content but those others far away have close to zero is satisfied.

Once impacts occur, the power of each sensor signal can be calculated in a chosen time window using Equation (10). When the power value is calculated at each sensor position in a structure, a smooth power distribution with minimum curvature can be found by the Robust Lowess Smoothing method, and then the smoothed power distribution can be expressed in terms of *x* and *y* coordinates, as follows:

(11) $$P_{i}=P_{i}\left(x_{i},y_{i}\right)\;i=1,\;2,\;\ldots$$

Using the smoothed power distribution found in Equation (11), an impact location can be estimated in terms of the centroid of the corresponding pulse power. In the *x* and *y* coordinates system of a structure, the centroid can be given by:

(12) $$x_{c}=\frac{\sum^{\text{​}}P_{i}∙x_{i}}{\sum^{\text{​}}P_{i}}\;y_{c}=\frac{\sum^{\text{​}}P_{i}∙y_{i}}{\sum^{\text{​}}P_{i}}$$

where $x_{c}$ is centre of the *x* axis, and $y_{c}$ is centre of the *y* axis.

In implemented experimental tests, a CFRP cantiliver structure was used to verify that this method provides a reliable and effective way to isolate an impacted region made up of sensor arrays, regardless of the surrounding noise contamination conditions.

#### 2.3.2. Locating Impact Coordinates

To update the accurate locations of impact forces acting on a structure, there exists an effective search parameter index—Time of Flight (*ToF*), which is an important characteristic parameter that represents how the stress waves propagate in a structure.

**Figure 6:**
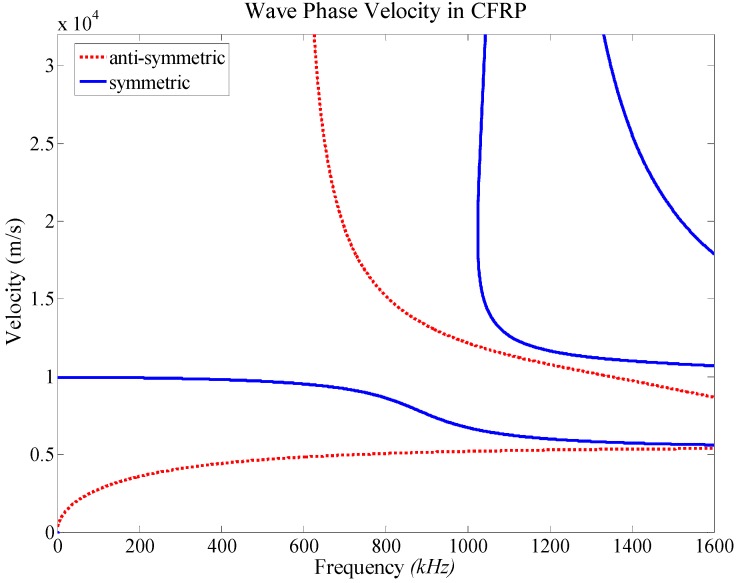
An example of phase velocity of propagating wave in CFRP.

Within an identified impact zone which is composed of four neighboring sensors, using the initially assumed wave propagation angles ${\text{θ}}_{i}$, the initial distance $L_{i}$ between unknown impact position and every sensor $S_{i}$ can be calculated firstly using Equation (13), and four initially estimated impact positions resulting from $L_{i}$ are also obtained simultaneously. Then a quadrilateral is composed of those positions. To obtain finally the accurate impact coordinates, a procedure of minimizing the area of the dashed quadrilateral must be executed, shown as Figure 6.

**Figure 7:**
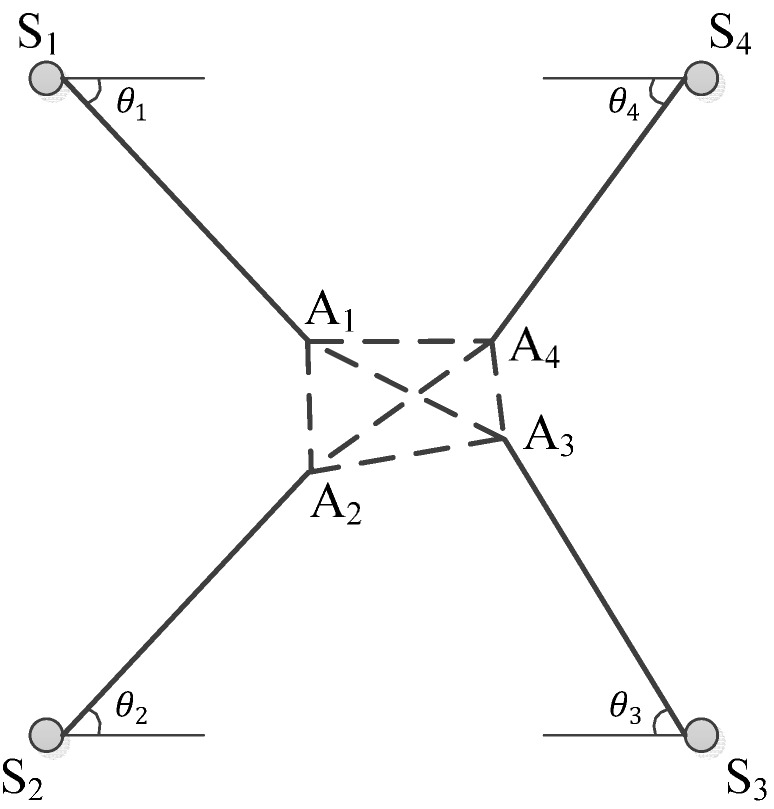
Demonstration of impact positioning procedure through updating quadrilateral.

A final estimation for the unknown impact coordinates can be calculated by the principle of quadrilateral centroid, which is expressed in Equations (15) and (16):

(13) $$L_{i}=C_{p}\left({\text{θ}}_{i}\right)\times ∆T_{i}\;i=1,\;2,\;3,\;4$$

where $C_{p}\left({\text{θ}}_{i}\right)$ is the phase velocity of stress waves [23,24] with the deflection angle of the propagating path from the impact to sensor $S_{i}$ , as shown in Figure 7.

$∆T_{i}$ is defined as the Time of Flight in Equation (14), that is, the time difference between the time an impact force acts and the Time of Arrival (*ToA*) recorded by a specific sensor:

(14) $$∆T_{i}=\left|t_{a}-t_{f}\;\right|\;$$

where $t_{a}$ is the time of arrival recorded by the specific sensor, and $t_{f}$ is the time of action of the impact force from the reconstructed force time history.

**Figure 8:**
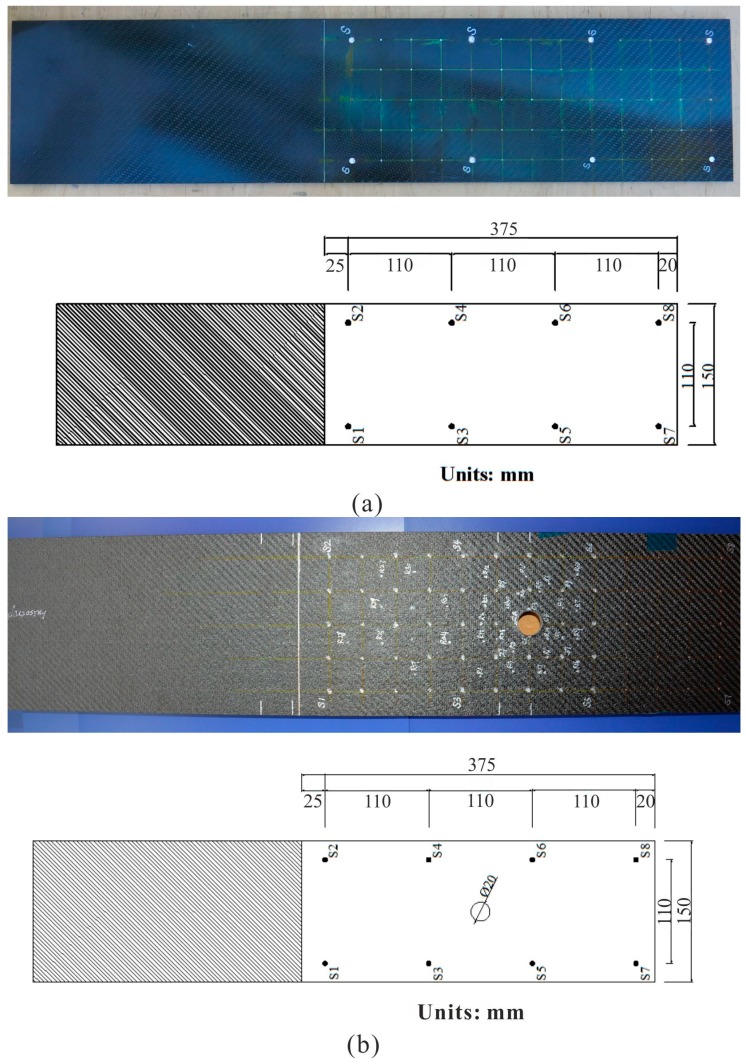
Geometries of the CFRP specimens and their sensor locations. (**a**) Specimen 1; (**b**) Specimen 2.

Meanwhile, four different angles (namely ${\text{θ}}_{1}$, ${\text{θ}}_{2}$, ${\text{θ}}_{3}$, ${\text{θ}}_{4}$) for wave propagation directions are assumed. Additionally, $∆T_{i}$ is the time of flight for sensor $S_{i}$:

(15) $$X_{imp}=\frac{1}{6M}\overset{4}{\underset{i=1}{\sum }}\left(x_{i}+x_{i+1}\right)\left(x_{i}y_{i+1}-x_{i+1}y_{i}\right)$$

(16) $$Y_{imp}=\frac{1}{6M}\overset{4}{\underset{i=1}{\sum }}\left(y_{i}+y_{i+1}\right)\left(x_{i}y_{i+1}-x_{i+1}y_{i}\right)$$

where *M* is the area of the quadrilateral, $M=\frac{1}{2}\overset{4}{\underset{i=1}{\sum }}\left(x_{i}y_{i+1}-x_{i+1}y_{i}\right)$. $x_{i}$ and $y_{i}$ are the x and y coordinates for the vertices $A_{i}$ (*i* = 1, 2, 3, 4), respectively. The entire procedure is presented graphically in Figure 8, and can be implemented for any quadrilateral sensor network configuration.

## 3. Experimental Details and Procedure

With the theoretical development and computer implementation of the IVI technique, it is necessary to set up experimental tests for some specific practical applications, in order to validate the IVI technique’s effectiveness and practicality. Thus, experimental tests were performed on two CFRP panel structures with different impact conditions and various surrounding noise environments. Among them, Specimen 1 is a normal CFRP plate, and Specimen 2 is a CFRP panel with an “orange peel” surface and a cutout hole.

### 3.1. Experimental Specimens

Specimen 1 (Figure 8a) is a CFRP plate with 660 by 150 by 5 mm^3^ size, and its layups are adopted as [0°, 90°]_w_/[0°/90°]_8s_/[0°, 90°]_w_. Meanwhile, the sensor spacing is chosen as 110 by 110 mm^2^, as shown in Figure 8a. Specimen 2 is similar to Specimen 1, and it has the same size and layups as Specimen 1, but it is an inhomogeneous structure with an “orange peel” surface and in addition, it has a circular cutout hole in the plate, as shown in Figure 8b.

Two distributed piezoelectric sensory networks and the associated wiring were mounted individually on the backward surfaces of the two CFRP panel structures, which are considered from the perspective of practical engineering demands and are also convenient to produce impacts on the forward surfaces of two CFRP panels by an instrumented hammer and several small balls. In order to ensure good surface conduction at the sensor locations, the surfaces of the two CFRP panels were sanded and a small amount of conductive epoxy was applied.

### 3.2. Experimental Setup

The two CFRP panel structures were impacted by a hand-held, instrumented hammer manufactured by PCB Piezotronics (Depew, NY, USA). Both the force signals from the impact hammer and the sensor output data from the structural responses were recorded using a computer data acquisition (DAQ) system. Also, a function/arbitrary waveform generator and a power amplifier were utilized to generate a series of repetitive random interfering noises. In addition, the sampling rates from 20 to 50 kHz were collected and all data were in the range of ±10 V.

**Figure 9:**
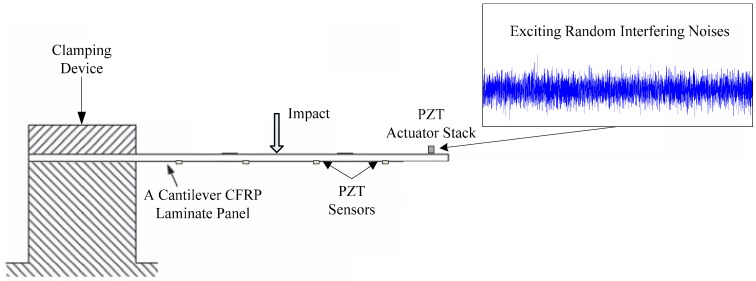
Boundary and environmental conditions for Specimen 1.

The two specimens were used to verify the established adaptive forward models, to assess impact location estimations, and to evaluate the applicability of the IVI technique due to the diversifications of composite materials, structure configurations, the types of impactors and environmental conditions, for example, random interfering noises. Meanwhile, Specimens 1 and 2 were fixed on one side and the other side was free, built as cantilever structures. Here, only the structure configuration for Specimen 1 is presented in Figure 9.

### 3.3. Impact Tests

**Figure 10:**
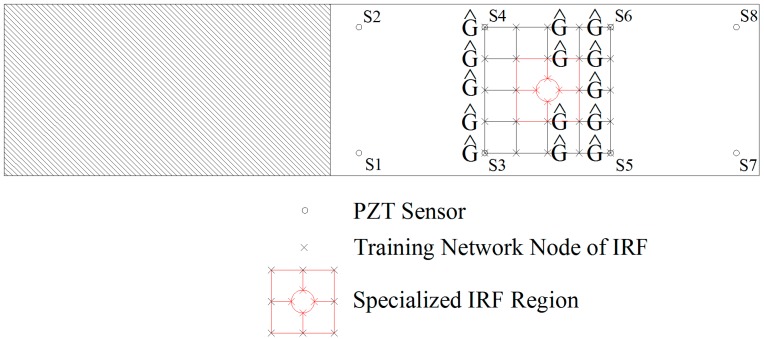
Demonstration for the formation of IRF network on Specimen 2.

In order to obtain the various IRFs, impact experimental tests were performed on the two specimens. For forming the network nodes of the various IRFs, force signals and sensor signals from the structural responses were recorded, (1) from 65 impact test points on Specimen 1; (2) from 68 impact test points on Specimen 2 (the schematic demonstration is shown in Figure 10), where the experimental impacts were made at evenly spaced locations, and random force magnitude was applied at each location.

**Figure 11:**
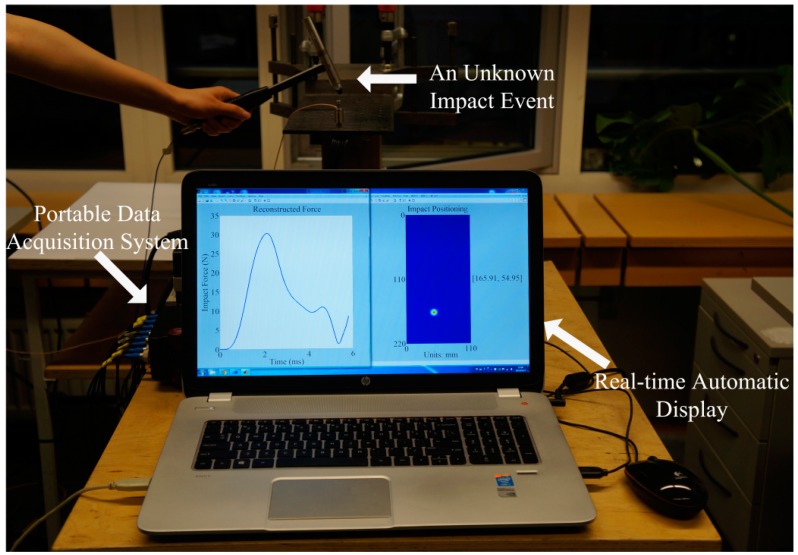
Real-time visualization inspection for an unknown impact event.

Once the corresponding network made up of the IRF nodes of Specimen 1 and 2 were found individually, various experimental impact tests could be made on any arbitrary location of either Specimen 1 or 2. A demonstration with an unknown impact event on Specimen 1 is illustrated in Figure 11 to indicate the IVI technique online visualization and automation. Also, a series of verifications of the impact identification were effectively implemented.

## 4. Results and Discussion

The estimated and reconstructed results are illustrated to validate the efficacy of the IVI technique for impact source identification. Then, the issues concerning the assessments of estimated impact locations, the effects of various anisotropic panel structures on force reconstruction, the effects of different supporting structures on the force reconstructions and the effects of surrounding noise contamination on force reconstructions will be discussed in detail.

### 4.1. Impact Positioning and Error Evaluations

**Figure 12:**
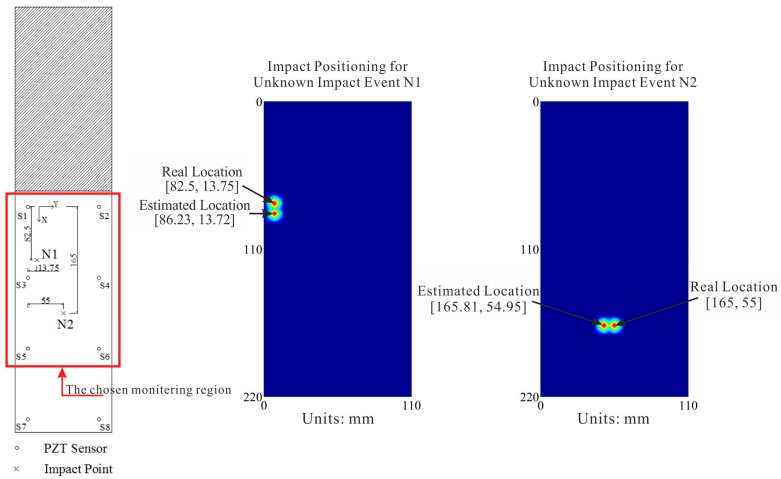
Estimations of impact locations for two unknown impact events on the normal CFRP structure.

**Figure 13:**
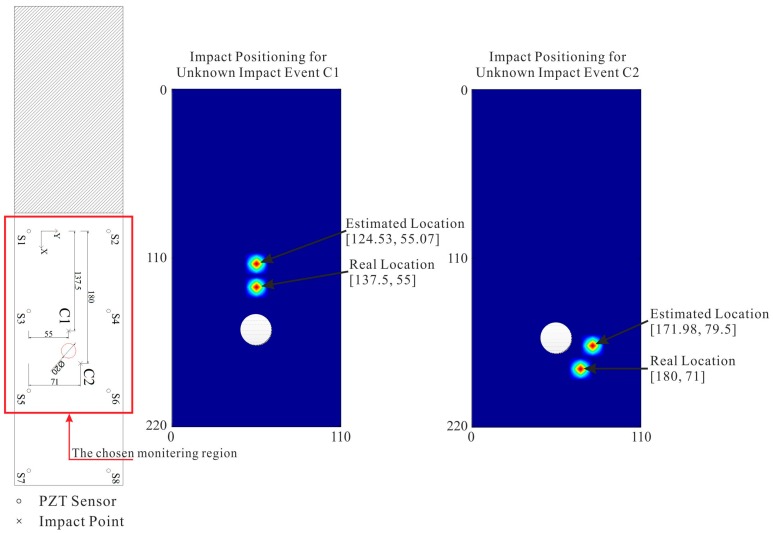
Estimations of impact locations for two unknown impact events on the cutout CFRP structure.

To illustrate the proposed monitoring and identification scheme, Figure 12 presents a set of estimated results for impact locations on Specimen 1, and a set of estimated results for impact locations on Specimen 2 are shown in Figure 13.

Afterwards, in order to assess the estimated impact locations, the evaluated location error is defined in Equations (17)–(19), which is expressed as the distance from the calculated impact location to the actual impact location. Here, for Specimens 1 and 2, the *x*-direction is defined as the length direction of the CFRP plate:

(17) $$\text{Δ}x=x_{cal}-x_{act}\;$$

(18) $$\text{Δ}y=y_{cal}-y_{act}\;$$

(19) $${\text{ζ}}_{e}=\sqrt{\text{Δ}x^{2}+\text{Δ}y^{2}}$$

**Figure 14:**
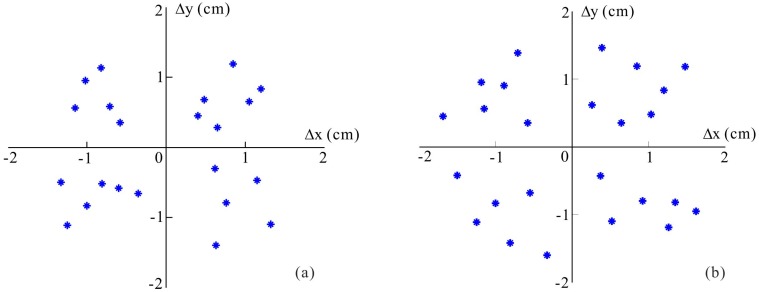
Location estimation errors for the random impact events. (**a**) On the normal structure; (**b**) On the cutout structure.

Through a series of impact verification tests on Specimen 1 and 2, generally the average location errors are mostly in the ranges of 10.3% and 12.3% of the corresponding mounted sensor spacing respectively, as shown in Figure 14.

### 4.2. Impact Identifications

**Figure 15:**
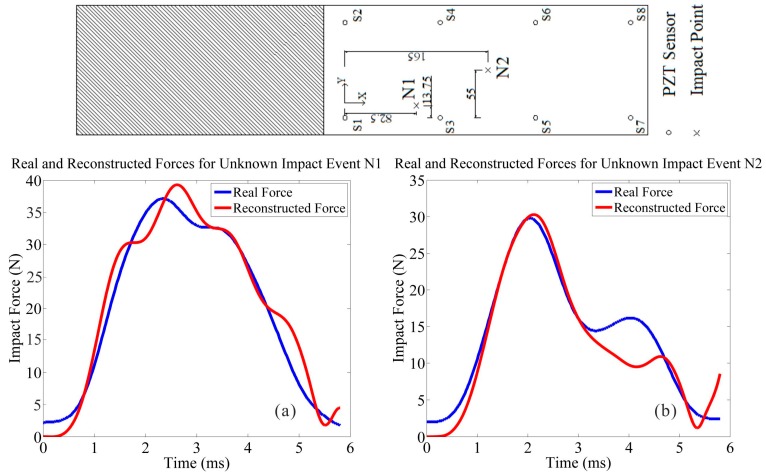
Impact identifications on the normal CFRP structure (Specimen 1). (**a**) Impact identification for event N1; (**b**) Impact identification for event N2.

**Figure 16:**
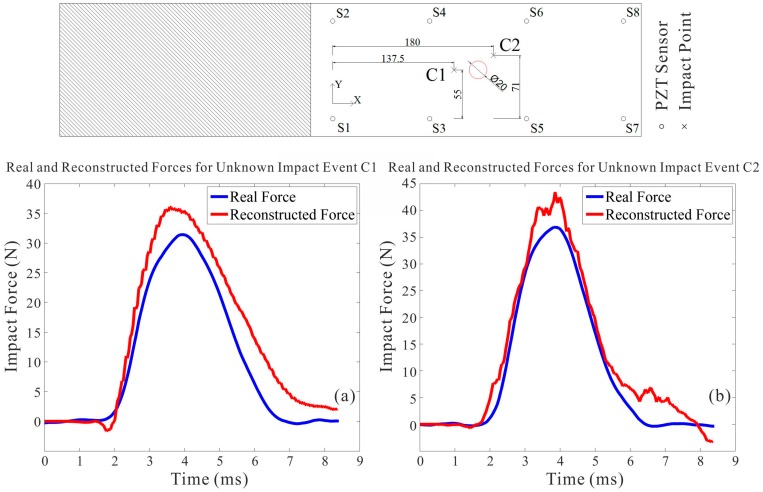
Impact identifications on the cutout CFRP structure (Specimen 2). (**a**) Impact identification for event C1; (**b**) Impact identification for event C2.

The impact identification function of the IVI technique was validated from two aspects that are diverse structure configurations and vibration noises contamination. The validation details and the relevant discussion are described in this section:

(1) To validate the efficacy of the impact identification function, a series of impact tests were implemented on the various CFRP panel structures (Specimens 1 and 2). However, in order to reveal the impact identification performance of the IVI technique comprehensively, some impact events with representative impact locations are selected and illustrated in Figure 15 and Figure 16. For Specimen 1, impact event N1 is selected due to the consideration of boundary performance validation, and impact event N2 is selected with the consideration of general performance validation. As well, for Specimen 2, two impact events C1 and C2 which both occurred at surrounding positions close to the cutout hole of the structure were chosen. The impact event C1 occurred in the vicinity of the upper boundary of the structure’s discontinuity, and the impact event C2 occurred in the vicinity of the lower boundary of the structure’s discontinuity. The two impact events on a cutout structure were selected to verify the anti-discontinuity capability of the impact identification function due to the inhomogeneous property of the structure, typically, an airplane fuselage panel with a window cutout frame.

(2) Nevertheless, to verify the impact identification capability against vibration noise effects, impact tests under changing random interfering noise conditions were implemented. Two representative changing random vibration disturbance conditions were selected, where one is under the noise condition of signal-to-noise ratio (*SNR*) of 15 and the other one is under the noise condition of SNR of 10.

Then through the identification process using the IVI technique, the different impact identification results are indicated in Table 1 and compared to confirm the resistibility performance for disturbances, where include that the identification results under de-noising, the identification results using vibration noises of *SNR*=15 and with vibration noises of *SNR* = 10.

**Table 1:** Impact evaluation results with vibration noises of *SNR* = 15 and 10.

Impact Events		Real	Estimations by Denoising	Estimations within Noises
SNR15	Maximum amplitude (Unit: N)	43.28	44.35	49.62
	Impulse (Unit: N s)	0.0925	0.1089	0.1295
SNR10	Maximum amplitude (Unit: N)	18.84	18.38	23.83
	Impulse (Unit: N s)	0.0616	0.0543	0.0893

With the IRF matrix networks established, the identification results are calculated, and they match well with the actual impact forces recorded in terms of maximum amplitude, force duration and impulse. Furthermore, in order to assess the accuracies of the identification results, the relative average errors $e_{a}$ corresponding to the above three parameters are necessary to be compared by using the following equation:

(20) $$e_{a}=\frac{1}{n}\overset{\text{n}}{\underset{\text{i}=1}{\sum }}\frac{V_{e}-V_{r}}{V_{r}}\times 100\%\;n=1,\;2,\;\ldots$$

where $V_{e}$ is the estimated value, $V_{r}$ is the actual value.

In view of all the impact tests performed, as for the normal structure (Specimen 1) without any vibration disturbance, the corresponding average error of the maximum amplitude is 7.8%, the corresponding average error of force duration is 12.8%, and the corresponding average error of impulse is 11.3%; as for the cutout structure (Specimen 2) with the inhomogeneous property, the corresponding average error of maximum amplitude is 8.3%, the corresponding average error of force duration is 13.9%, and the corresponding average error of impulse is 12.5%; additionally, for the structure under random vibration conditions, through the de-noising process, the corresponding average error of the maximum amplitude is 8.1%, the corresponding average error of force duration is 13.3%, and the corresponding average error of impulse is 11.8%. However, when the overall average errors of impact identifications with noise contaminations are calculated out, they are both a little more than the results of the de-noising implementation. In general, with output noise signals of *SNR* = 15 (with noise contamination), the overall average error in impact identification is approximately 6.9% more than that of de-noising implementation; and with output noise signals of *SNR* = 10 (with noise contamination), the overall average error in impact identification is approximately 8.6% more than that of de-noising implementation.

As a result of the relative comparisons of all estimated results, it is easy to see that the results of impact identifications obtained by de-noising processing are better than those identification results with noise contaminations. However, this causes the above situation of different errors, the main reason for which is that there exist several disturbed and unstable factors that are known or unknown resulting from vibrations while the experimental tests are being performed, for instance, nonlinear problems, vibration randomness problems, unpredictable impact conditions and the effect of stress wave propagation due to vibration, *etc*.

### 4.3. Structural State Awareness

**Figure 17:**
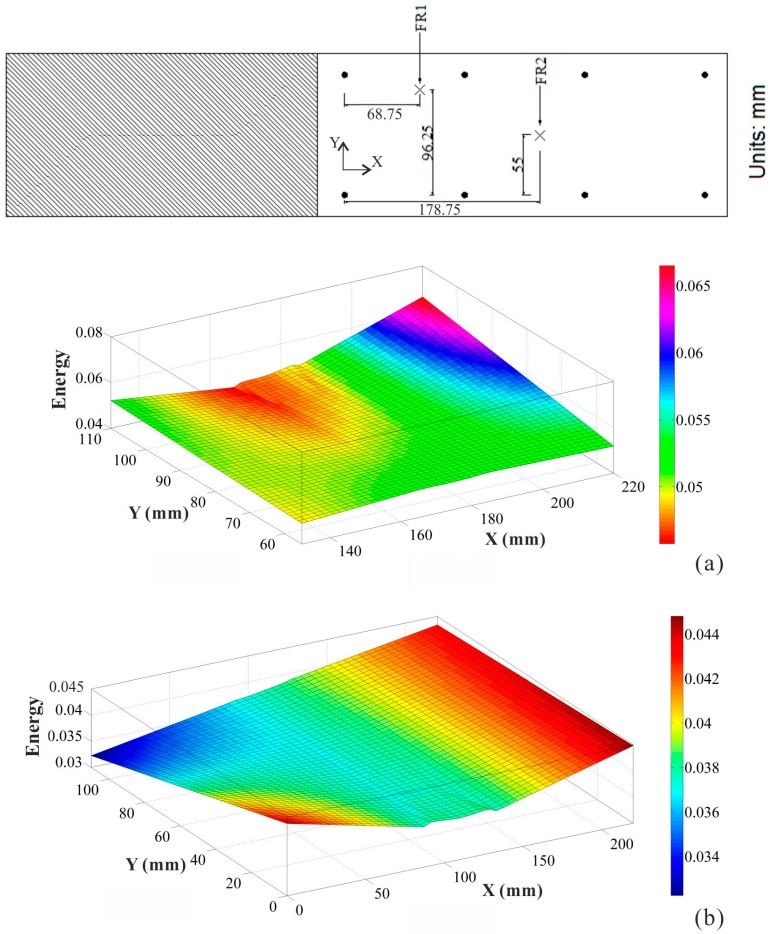
Structural state monitoring and assessment when impact events occurred. (**a**) Structural state when impact at location FR1; (**b**) Structural state when impact at location FR2.

What happens in the structure when impact events occur unexpectedly on a structure? How does the structural state alter? And what effects are produced in the structure? With a set of the above problems, an approach of the absorbing energy distribution in a structure provides a better solution to solve the intractable problems met, which is resulting from any impact event. Through the absorbing energy distribution (AED) method, the structural state can be monitored in real-time mode. Also, the AED method can analyze and evaluate using the output data from the structural response to supply a demonstration for determining the corresponding FE model order generated due to any impact event. Accordingly, Specimen 1 was used as this demonstration to illustrate the synthesized performance of the impulse energy distribution method, which is presented in Figure 17.

## 5. Conclusions

This paper presents a systematical impact inspection and structural condition monitoring scheme. By using the distributed sensors networks defined, an advanced real-time IVI technique is proposed to estimate the impact locations and force histories including the information about the force magnitudes and to visualize the structural state when impacts occurred. In the automatic identification procedure, a precise forward model for a given structure can be established rapidly through the functional module of forward model generator. Meanwhile, by incorporating with the dynamic state-space model, the impact forces can be reconstructed from a small number of impulse response functions from the optimal parameters ($a_{i},b_{j}$) of the model structure calculated using the proposed fast GA parameter estimation during forward modeling, where eliminates the need of numerous impact training tests for constructing the network of impulse response functions on a structure. Nevertheless, in the impact positioning and evaluation procedure, the initial impact locations can be estimated using the developed smoothed power distribution method, afterwards on the basis of the results from the initial location estimations, the accurate location coordinates of each impact can be updated further by the proposed time of flight (ToF) based quadrilateral sensor network positioning method. It is worth noting that to achieve a highly reliable and highly robust impact monitoring and identification system adapting into complex and harsh engineering environments, the real-time IVI technique shows satisfactory success in predicting impact locations and estimating force histories for various types of structure configurations and under mutative vibration environment conditions, and its capability of structural state monitoring and real-time assessment is also verified. Through all cases of the impact tests considered, the impact visualization inspection technique shows its potential as an on-board rapid diagnostic tool of accidental impact events that can cause possible damage in an aerospace composite structure.
